# Dissecting trophoblastic heterogeneity in abnormal pregnancy: Insights from comparative analysis of twin-pregnancy with hydatidiform mole and coexisting live fetus

**DOI:** 10.1016/j.gendis.2025.101651

**Published:** 2025-04-28

**Authors:** Chen Li, Jiandong Chen, Hao Wu, Songfa Zhang, Na Yu, Zhiang Chen, Bingjian Lu, Santasree Banerjee, Weiguo Lu, Jiale Qin

**Affiliations:** a Department of Human Genetics, Women's Hospital, School of Medicine, Zhejiang University, Zhejiang Provincial Key Laboratory of Genetic and Developmental Disorders, Hangzhou, Zhejiang 310006, China; b Women's Hospital, School of Medicine, Zhejiang University, Hangzhou, Zhejiang 310006, China; c Zhejiang Key Laboratory of Precision Diagnosis and Therapy for Major Gynecological Diseases, Hangzhou, Zhejiang 310006, China; d Zhejiang Provincial Clinical Research Center for Gynecological Disease, Hangzhou, Zhejiang 310006, China; e Zhejiang Key Laboratory of Maternal and Infant Health, Hangzhou, Zhejiang 310006, China

Complete hydatidiform mole (CHM), a typical gestational trophoblastic disease (GTD), is a consequence of abnormal fertilization.^1^ Historically, trophoblastic cells have been categorized based on morphological and pathological characteristics, with functional classification yet to be well-established. Genetic abnormalities in these cells have been primarily studied using standard techniques such as short tandem repeats (STR) genotyping or microarray comparative genomic hybridization (aCGH).^2^ Previous studies using single-cell RNA sequencing (scRNA-seq) have uncovered the transcriptomic map of normal human placental cells.^3^, ^4^, ^5^ However, the key genes and transcriptional characteristics of trophoblast differentiation under pathological conditions remain largely unexplored due to genetic complexity and individual heterogeneity. We performed scRNA-seq on villus tissues freshly collected from two Chinese twin-pregnancies with CHM and coexisting live fetus (CHMCF) to compare the distinct transcriptional profiles. In both patients, STR analysis revealed that the molar tissues contained only paternal STR loci, confirming their identification as androgenetic monospermic CHM (Fig. 1A). In a twin pregnancy model of CHMCF, both the CHM and live fetus have the same gestational age (i.e., the timing of trophoblast differentiation) and are exposed to the same uterine environment. This theoretically identical developmental context provides optimal conditions for a comparative study.

**Figure 1:**
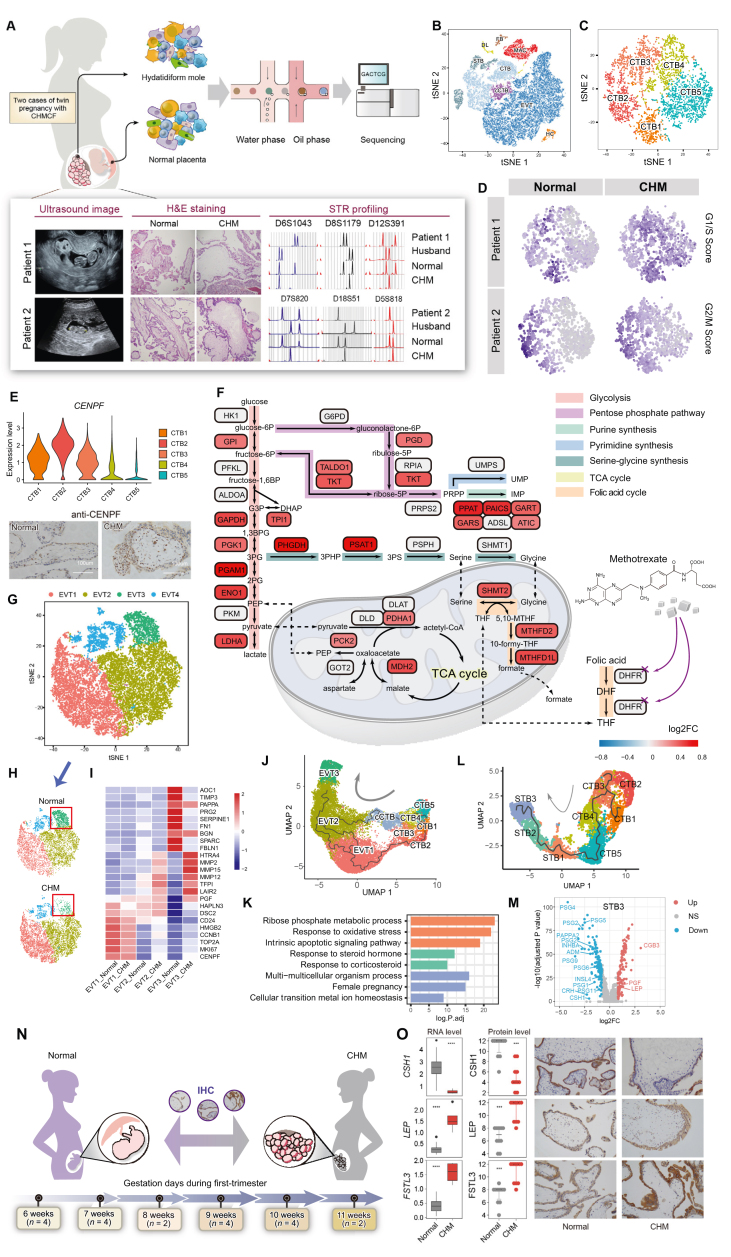
Single-cell expression atalas of CHMCF. **(A)** Two Chinese patients were diagnosed with twin pregnancy with CHMCF based on ultrasound findings, pathological evaluation, and cytogenetic testing. (**B)** tSNE projection of total cells, with clusters color-coded according to cell type: cytotrophoblast (CTB), syncytiotrophoblast (STB), extravillous trophoblast (EVT), column cytotrophoblast (cCTB), Hofbauer cell (HC), fibroblast (FB), macrophage (MAC) and decidual lymphocyte (DL). (**C)** tSNE plot showing a total number of 5139 CTB cells, re-clustered as CTB1, CTB2, CTB3, CTB4, and CTB5. **(D)** Distribution of cell cycle score across CTB cells from normal and CHM villi, respectively. **(E)** Gene expression levels of CENPF across CTB subtypes (upper). IHC staining of formalin-fixed and paraffin-embedded sections obtained from normal and CHM samples (lower); scale bar = 100 μm. **(F)** Differentially expressed genes in CTB1 cells (CHM versus normal villus) involved in the central carbon metabolic pathway, SGOC metabolism, and oxidative phosphorylation. **(G)** tSNE projection of 19,293 EVTs. Four clusters were identified. **(H)** Distribution of EVT subtypes between normal and CHM villi, derived from (G), showing a reduction in the total number of EVT3 in CHM. **(I)** Heatmap of the mean expression levels of up-regulated genes in the three subtypes of EVT. Values are scaled by mean-centering and transformed to a scale from −2 to 2. **(J)** UMAP projection and cell differentiation trajectory of cells using Monocle 3 trajectory analysis. **(K)** Bar plot of enriched GO terms for significantly up-regulated genes in STB1 and STB2 cells. **(L)** UMAP projection of cells obtained by combining CTB1–5 and STB1–3 cells from the trajectory analysis using Monocle 3, with the cell differentiation pathway and direction of differentiation indicated. **(M)** Volcano plot demonstrating differentially expressed hormone-encoding genes in STB3 cells in CHM. **(N)** Ten aborted normal fetuses and ten gestational age-matched CHMs were recruited for the validation study. **(O)** Box plots depicting the gene expression levels of selected hormone-encoding genes from bulk RNA-seq data (N_*normal*_ = 39, N_*CHM*_ = 4), and bee swarm plots of the quantified protein levels of the matched genes in the validation cohort (N_*normal*_ = 10, N_*CHM*_ = 10). ∗∗∗, *p*-value < 0.001; ∗∗∗∗, *p*-value < 0.0001. IHC staining of formalin-fixed and paraffin-embedded sections obtained from normal and CHM samples; scale bar = 100 μm.

After doublet filtering and quality control, a total of 32,989 cells were obtained. Eight cell types were identified (Fig. 1B) in both CHM and normal placenta, including four trophoblasts–cytotrophoblast (CTB), syncytiotrophoblast (STB), extravillous trophoblast (EVT), and column cytotrophoblast (cCTB), and four non-trophoblasts - Hofbauer cell (HC), fibroblast (FB), macrophage (MAC), and decidual lymphocyte (DL). We re-clustered the CTB cells to five subtypes (Fig. 1C). In both patients, we observed significant CTB3 population expansion and CTB5 population contraction in CHM. The expressions of marker genes *RRM2*, *PCNA*, and *TOP2A* were all highly correlated with cell proliferation in CTB1-CTB3. The cell-cycle stage analysis indicated that CTB1 and CTB3 were in the G1/S phase, while CTB2 was in the G2/M phase (Fig. 1D). Gene Ontology (GO) analysis revealed that CTB1-CTB3 cells were enriched for DNA replication, nuclear division, and ribonucleoprotein complex biogenesis, suggesting that CTB1 might be S-phase-like and CTB3 is G1-phase-like cell populations (Table S1). The expression of the cell cycle-associated nuclear antigen CENPF was also observed to be higher in CTB1 and CTB3, and highest in CTB2 (Fig. 1E). Immunohistochemistry (IHC) staining validated a significant increase in CENPF-positive CTB cells in CHM, indicating that CTB1-CTB3 in CHM are associated with the processes of cell cycle and proliferation. Gene set variation analysis (GSVA) revealed that metabolism were significantly upregulated in CHM compared to normal villi (Fig. S1 and Tables S2, S3).

Glycolysis/gluconeogenesis pathways were more active in CHM, with significant upregulation of glycolysis enzyme genes in CTB3 (Fig. 1F). However, *HK1, PFKL* and *PKM* genes showed no significant difference. The upregulation of mitochondrial phosphoenolpyruvate carboxykinase (PCK2) in CHM indicates that gluconeogenesis pathway, promoted by the upregulation of PCK2, is primarily responsible for the enhanced glycolysis/gluconeogenesis activity in CHM.

The non-oxidative phase of the pentose phosphate pathway (PPP) was also upregulated in CHM. *TALDO1* and *TKT* genes, involved in this phase and ribulose-5-phosphate supply, were upregulated, facilitating nucleotide synthesis. The *de novo* purine biosynthetic pathway replenishes the purine nucleotides during high demand, such as cell division and tumor proliferation. We observed that, five enzymes—GART, GARS, PAICS, ATIC and PPAT—showed upregulated expressions in CHM. This suggests an elevated requirement for purine nucleotides in CHM, likely aiding the excessive proliferation through increased activity of the PPP and purine synthesis pathways.

We observed a significant increase in the expression of genes encoding key enzymes of the serine–glycine one carbon (SGOC) pathway, *PHGDH* and *PSAT1*, and a notable overexpression of the mitochondrial enzymes *SHMT2*, *MTHFD2*, and *MTHFD1L*, which are involved in the folate cycle, in CHM compared to normal villi. SHMT2 produces 5,10-methylenetetrahydrofolate (5,10-MTHF) for nucleotide biosynthesis. MTHFD2 and MTHFD1L supply one-carbon units for metabolism, which are essential for rapid cell growth and proliferation. Interestingly, their cytoplasmic isoforms (SHMT1/SHMT2α and MTHFD1) showed no expression change.

We re-clustered EVT cells into four subtypes (EVT1-4) (Fig. 1G). EVT1 highly expressed canonical cell-cycle related genes and adhesion related marker genes. EVT2 was characterized by matrix metallopeptidases (MMPs) associated with extracellular matrix (ECM) degradation, with higher expression in CHM. In EVT3, the differentially expressed genes (i.e., marker genes) were primarily associated with ECM organization and inhibition of its degradation, which were notably absent in CHM (Fig. 1H; Fig. S2 and Table S2). The higher expression levels of ECM degradation genes in EVT2 and the absence of EVT3 indicate that EVT cells in CHM induce higher ECM degradation and display over-invasive characteristics (Fig. 1I). Trajectory analysis suggested that EVTs originated from both CTB3 and cCTB, with both subtypes differentiated into EVT3 (Fig. 1J). It is speculated that EVTs from CHM tissue exhibit a delayed expression pattern compared to normal EVTs. GSVA analysis revealed disrupted TNF-α, TGF-β, and IFN-γ signaling pathways in CHM, indicating impaired cell differentiation and increased invasiveness of EVTs (Fig. S1 and Table S2). These findings highlight the EVT invasiveness in CHM and suggest potential regulatory mechanisms of progression to the invasive stage.

STBs are crucial for hormone secretion during pregnancy, but the specific STB subtypes responsible for the expression of these hormone-encoding genes remain unclear. From 2090 STBs, we identified four subtypes. We observed that *ERVFRD-1*, a gene associated with CTB syncytisation, was highly expressed in STB1 in both normal and CHM villi. *PAGE4* and *PEG10*, both CTB markers, were highly expressed in STB1, suggesting that STB1 was in the early stage of STB differentiation. GO and GSEA revealed that STB1 was enriched for apoptosis pathways (Fig. 1K and Tables S1, S2), suggesting that apoptosis may promote CTB fusion and STB differentiation. STB2 represented an intermediate state between immature and mature STBs, with high expression of STB1 and STB3 maker genes (Fig. S3). Trajectory analysis predicted that G1 phase CTB3 cells transition to a non-proliferative state (CTB4/CTB5), followed by the progression to immature STB1 and ultimately to STB3 cells, the terminal stage of differentiation (Fig. 1L). Therefore, STB3 cells were considered mature and responsible for hormone synthesis and secretion.

We also observed significant differences in hormone-encoding genes (HEGs) in STB3 between normal and CHM villi. Of the 48 HEGs, three genes (*CGB3*, *LEP*, and *FSTL3*) were upregulated in CHM, while 14 were downregulated (*p*-value<0.01, |logfc|>0.8) (Fig. 1M), which was further validated using external bulk RNA-seq datasets (GSE109082 and GSE138250). Statistically significant differences were observed in the expression of *CSH1* (encoding hPL), *LEP* (encoding adipocyte hormone LEPTIN), and *FSTL3* (encoding follistatin-related protein) between normal and CHM villi (*p*-value<0.05). These differences were further validated using IHC staining in 10 pairs of matched surgical specimens from aborted normal embryos and CHM tissues (6–11 weeks gestation) (Fig. 1N). Protein expression levels of three significantly expressed HEGs were fully consistent with the results of both the bulk RNA-seq and scRNA-seq analyses (Fig. 1O).

Collectively, this study represents a comprehensive investigations of gene expression profiles in CHMCF, shedding light on the transcriptional landscape of abnormal pregnancy. By identifying molecular signatures, it highlights their potential as early diagnostic markers for trophoblast-related pregnancy abnormalities.

## CRediT authorship contribution statement

**Chen Li:** Writing – review & editing, Writing – original draft, Software, Funding acquisition, Formal analysis, Data curation, Conceptualization. **Jiandong Chen:** Writing – original draft, Visualization, Software, Formal analysis, Data curation. **Hao Wu:** Visualization, Software, Investigation, Formal analysis, Data curation. **Songfa Zhang:** Validation, Resources, Investigation, Data curation. **Na Yu:** Writing – original draft, Validation, Resources, Investigation, Data curation, Conceptualization. **Zhiang Chen:** Visualization, Software, Formal analysis, Data curation. **Bingjian Lu:** Validation, Resources, Data curation. **Santasree Banerjee:** Writing – review & editing, Validation, Investigation. **Weiguo Lu:** Writing – review & editing, Supervision, Resources, Project administration, Funding acquisition, Conceptualization. **Jiale Qin:** Writing – review & editing, Writing – original draft, Validation, Supervision, Resources, Project administration, Methodology, Investigation, Conceptualization.

## Ethics declaration

The study was approved by the Ethics Committee of Women's Hospital, Zhejiang University School of Medicine (Approval number: IRB-20210167-R), and informed consent has been obtained from the patient.

## Data and code availability

The raw scRNA-seq data have been uploaded to National Genomics Data Center (NGDC) of China under accession number HRA003869 (https://ngdc.cncb.ac.cn/gsa-human/browse/HRA003869 for review).

## Funding

This work was supported by grants from the National Natural Science Foundation of China (No. 82272126 to CL); Key Research and Development Program of Zhejiang (No. 2023C03037 to WL) and National Key Research and Development Program of China (No. 2023YFC2705801 to JQ). Li C was supported by Alibaba Cloud.

## Conflict of interests

The authors declare no competing interests.
