# Supplementary material for: Dissecting trophoblastic heterogeneity in abnormal pregnancy: Insights from comparative analysis of twin-pregnancy with hydatidiform mole and coexisting live fetus
Source: Genes Dis. 2025 Apr 28;13(1):101651. doi: 10.1016/j.gendis.2025.101651 (PMC12624585; doi:10.1016/j.gendis.2025.101651)
Supplement: Multimedia component 1 [file mmc1.docx]

**SUPPORTING INFORMATION**

**Dissecting Trophoblastic Heterogeneity in Abnormal Pregnancy: Insights from Comparative Analysis of Twin-Pregnancy with Hydatidiform Mole and Coexisting Live Fetus**

Chen Li, Jiandong Chen, Hao Wu, Songfa Zhang, Na Yu, Zhiang Chen, Bingjian Lu, Santasree Banerjee, Weiguo Lu, Jiale Qin

Clinical characteristics of two Chinese twin pregnancies with CHMCF

We prospectively recruited two cases of twin pregnancies with CHMCF.

**Patient 1**: A 22-year-old pregnant woman (gravida 0 para 0) underwent artificial insemination for assisted reproduction. Pregnancy was confirmed 13 days after fertilization (4 weeks of gestation). She experienced symptoms such as vaginal bleeding, severe nausea, and vomiting. At 11 5/7 weeks of gestation, her tests showed a serum β-hCG level of 313,694 IU/L; alanine aminotransferase, 100U/L; and aspartate aminotransferase, 52 U/L. Hyperemesis gravidarum was present and ketone bodies (+++) were detected. On the same day, ultrasound scan showed a live embryo with a crown-rump length of 5.1 cm and a vesicular lesion (4.0×3.5×2.5 cm) beside the embryo sac (Figure 1A). Pregnancy was terminated at 12 2/7 weeks of gestation due to concerns of potential pregnancy-related complications (such as abnormal liver function). Pathological examination of the operative specimen revealed early placenta and CHM. Her serum β-hCG level declined to 3847 IU/L one week after evacuation.

**Patient 2**: A 34-year-old pregnant woman (gravida 3 para 1) underwent *in vitro* fertilization and embryo transfer due to salpingemphraxis sterility. She also presented with vaginal bleeding following conception. At 10 2/7 weeks of gestation, her serum β-hCG level was 924,904 IU/L; urine protein, positive; hemoglobin (Hb), 82 g/L; and blood pressure, 140/80 mmHg. On the same day, the ultrasound scan showed a live embryo with a crown-rump length of 4.0 cm and a vesicular lesion (10.4×5.1×9.8 cm) beside the embryo sac (Figure 1A). Pregnancy was terminated at 10 6/7 weeks of gestation due to concerns of potential pregnancy-related complications (such as hypertension). The tissues were pathologically diagnosed as early placenta and CHM.

## Methods and materials

### Tissue collection

Tissue samples from two pairs of twins who underwent elective termination in the first trimester were obtained via manual vacuum aspiration. Specimens sized 1 cm^3^ were isolated from the hydatidiform mole and normal chorionic villi and washed with phosphate-buffered saline (PBS) to remove maternal blood cells. These samples were immediately transferred for single-cell isolation and RNA-seq analysis. In addition, some specimens of the hydatidiform mole, chorionic villi, and fetal tissue were identified microscopically and embedded in paraffin. Finally, hydatidiform mole tissue, fetal tissue, and peripheral blood from the mother and father were collected for genome sequencing and short tandem repeat (STR) profiling.

### DNA extraction, PCR amplification, and STR typing

Genomic DNA was extracted from peripheral blood samples or tissue samples using the Chelex-100 method. Twenty-one autosomal STR loci (D19S433, D5S818, D21S11, D18S51, D6S1043, D3S1358, D13S317, D7S820, D16S539, CSF1PO, Penta D, vWA, D8S1179, TPOX, Penta E, TH01, D12S391, D2S1338, FGA, D2S441, and D10S1248) and one sex locus, Amelogenin, were amplified using the Goldeneye™ 20A kit or AGCU EX22 kit on a GeneAmp PCR System 9700 (Life Technologies, Foster City, USA) according to the manufacturer’s instructions. PCR products were detected using the ABI PRISM 3130xL Genetic Analyzer (Life Technologies). Allele identification and data analysis were performed using GeneMapper® ID v3.2 software (Applied Biosystems, Foster City, CA, USA). Control DNA 9947A was used as the positive control and ddH_2_O was used as the negative control for each batch of genotyping.

### Preparation of single-cell suspensions

After each specimen was washed in PBS and cut into 0.2-mm pieces, it underwent two steps of digestion. First, the specimen was digested for 7 mins at 37°C in RPMI-1640 medium (Gibco) containing 0.25% trypsin (Sigma-Aldrich) and 0.02% EDTA and then centrifuged at 300 x*g* for 5 min at room temperature (25°C). The supernatant was collected and mixed with an equivalent volume of 10% fetal bovine serum (FBS). Second, the remaining specimen was resuspended in RPMI-1640 medium and centrifuged at 300 x*g* for 5 min to obtain a pellet, which was then treated with collagenase I (1.0 mg/ml, Sigma-Aldrich) at 37°C for 10 min. An equivalent volume of 10% FBS was added to the supernatant. The cell suspension was obtained by merging the supernatant collected from the two steps above. Cells were filtered through 70–30-μm stacked cell strainers (BD Falcon) and collected following centrifugation at 300 x*g* for 5 min at 4°C. Cells were resuspended in 50 μL of 1× PBS containing 0.04% bovine serum albumin (BSA). Overall cell viability was assessed using the trypan blue exclusion method on a Countess II Automated Cell Counter. Following counting, the concentration of each suspension was adjusted to 700–1200 cells/μL.

### Single-cell capture, library preparation, and sequencing

Single-cell suspensions were loaded with 10x Genomics Chromium using the 10x Genomics Chromium Single-Cell 3’ kit (V3) to capture 10,000 single cells per sample according to the manufacturer’s instructions. cDNA amplification and library construction were performed subsequently according to the standard protocol. 10x Genomics-generated cDNA libraries were sequenced on an Illumina NovaSeq 6000 instrument at a targeted depth of 20,000 reads per cell (paired-end multiplexing run, 150bp). Finally, single-cell RNA-seq was performed by LC-Biotechnology Co. Ltd. (Hangzhou, Zhejiang, China).

### Single-cell RNA-seq data pre-processing and quality control

CellRanger pipeline, from the Chromium Single Cell 3’ Software Suite (10x Genomics), was utilized to process the raw sequencing data (BCL files), which were demultiplexed, aligned to the GRCh37/hg19 reference genome using STAR , and barcode processed to generate gene expression matrices. Quality control and downstream analysis were performed using the Seurat package (version 4.1.2) . We applied *DoubletFinder* to remove cell doublets from each sample individually . Potentially dead or low-quality cells were discarded if they did not meet the following criteria: mitochondrial reads ≤ 25% and 500–10,000 UMIs. Genes found to be expressed in >3 cells were retained.

### Clustering

All samples underwent the same normalization process and were integrated based on the canonical correlation analysis method using *FindIntegrationAnchors* and *IntegrateData* functions in Seurat. Following principal components analysis (PCA) of the integrated dataset, graph-based clustering was conducted using the *FindNeighbors* function according to the top 15 PCs and with the FindClusters function at a resolution of 1.4. Data visualization was performed using t-distributed stochastic neighbor embedding (tSNE). Each cell cluster was annotated for cell type based on known canonical markers. Only clusters with a median total count >3000 were used for subsequent analysis.

### Cell proportion analysis

The proportions of cell subtypes in the paired normal and CHM samples were compared using the Chi-square test and permutation testing was implemented using the scProportionTest R package (v1.0.0, <https://github.com/rpolicastro/scProportionTest>). Considering that the number of replicates was low (2 samples for each, normal and CHM), we applied the scCODA framework, which has proven capability in cases with few replicates, to additionally validate the compositional differences between normal and CHM conditions .

### Cell cycle analysis

A predefined gene set involved in the G1/S or G2/M phase was used. The cycling score of each cell was measured using the *AddModuleScore* function in Seurat. Cells with cycling scores above the threshold (the median cycling score + 2 🞨 median absolute deviation of cycling scores) were defined as cycling cells.

### Trajectory and pseudotime analysis

Monocle 3 was used to perform pseudotime analysis. The intergraded data were normalized using the *preprocess_cds* function . Subsequently, we performed PCA, uniform manifold approximation and projection (UMAP), and cell clustering using *reduce_dimension* and *cluster_cells* functions. Using the reduced dimension space, the *learn_graph* function was applied to learn the principal graph of input data for trajectory building. Pseudotime inference was carried out using the *order_cells* function to represent the degree of cell differentiation in the lineage.

### Pathway analysis

Data were predominantly processed to generate down-sampled and pseudo-bulk data by cell groups. Gene Set Variation Analysis (GSVA v1.42.0) was performed to examine pathway enrichment for 50 hallmark pathways and GO biological process pathways (MSigDB) . Differences between cell groups were calculated using the *limma* package (v3.50.1) .

### Bulk RNA-seq

Bulk RNA-seq data of the hydatidiform mole and normal placenta were downloaded from Gene Expression Omnibus (GEO) under accession numbers GSE138250 and GSE109082. Data were normalized as transcripts per million (TPM) and adjusted based on the expression of housekeeping genes.

### Validation cohorts

We retrospectively examined 10 cases of pathologically diagnosed CHM from the Biobank of Women’s Hospital, School of Medicine, Zhejiang University. The gestational ages of these CHM samples varied. The median age of the mothers was 27 years, ranging from 19 to 36 years. Each CHM was paired with a normal fetus of similar gestational age (< 3 days apart). The maternal age of the normal fetuses was 27.5 years (range 20 to 35 years). Fetuses were considered normal if the mother’s serum β-hCG value and fetal growth on ultrasound were consistent with gestational age. All normal fetuses were from elective pregnancy terminations. Fetuses were not included in the normal control group if abortion was inevitable; they had detectable anatomical abnormalities, nuchal translucency >2.5 mm, or karyotypic abnormality; or the mother had a history of pregnancy with chromosomal abnormalities. The gestational age of the 10 CHM-normal fetus pairs were 43-43, 46-47, 48-49, 50-51, 56-56, 65-62, 67-67, 70-70, 71-71, and 78-78 days.

### Hematoxylin and Eosin (H&E) staining and immunohistochemistry (IHC)

The formalin-fixed and paraffin-embedded (FFPE) tissue blocks were sliced into 4-µm sections, deparaffinized in xylene, rehydrated in graded ethanol, and stained with H&E solution. Immunohistochemistry was performed using an Envision detection system (Dako, Denmark) according to the manufacturer’s instructions. We used commercial antibodies against CENPF (ab84697, Abcam, 1:500), LEP (ab16227, Abcam, 1:8000), FSTL3 (ab232761, Abcam, 1:400), KISS1 (ab275874, Abcam, 1:5000), CRH (ab216599, Abcam, 1:2000), PSG9 (ab247069, Abcam, 1:2000), and CSH1 (ab15554, Abcam, 1:1500) for IHC. PBS was used as the negative control.

Five individual fields were selected for scoring each slide. Immunostaining intensity was classified into five grades: 0, negative; 1, weak; 2, moderate; 3, strong; and 4, very strong. The proportion of positively stained cells was also divided into five classes: 0, <5; 1, 6–25%; 2, 26–50%; 3, 51–75%; and 4, >75%. The IHC staining scores were obtained by multiplying the intensity score with the proportion score. All the staining results were evaluated independently by two well-trained observers blinded to the clinical data.


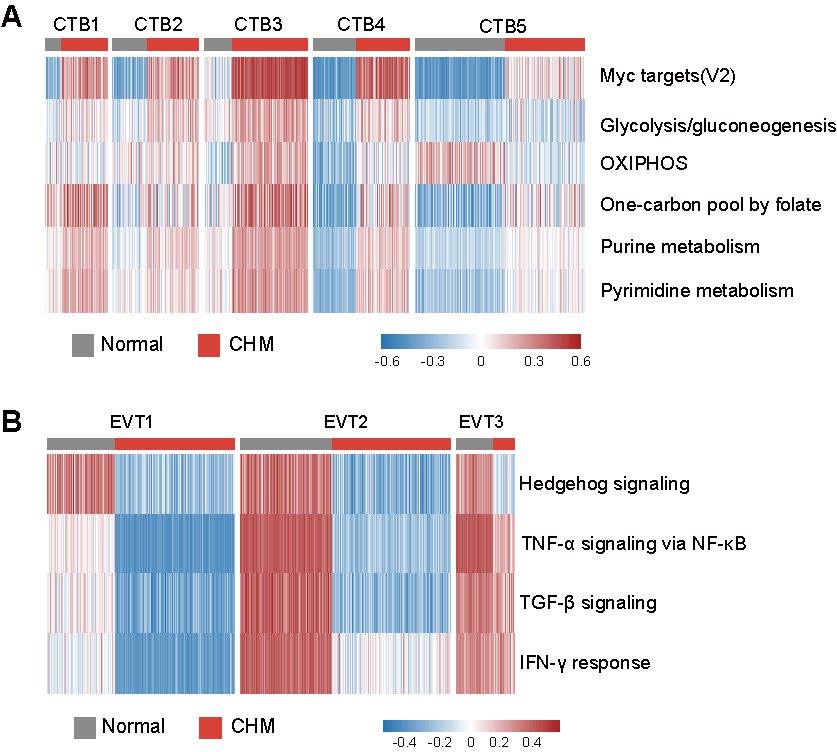


**Fig. S1** Supplementary results of GSVA analyses for CTB and EVT cell types. A. GSVA revealed that metabolism were significantly upregulated in CTB3 of CHM compared to normal villi. B. Significant disruption in TNF-α, TGF-β, and IFN-γ signaling pathways in EVT2 subtype of CHM patients.


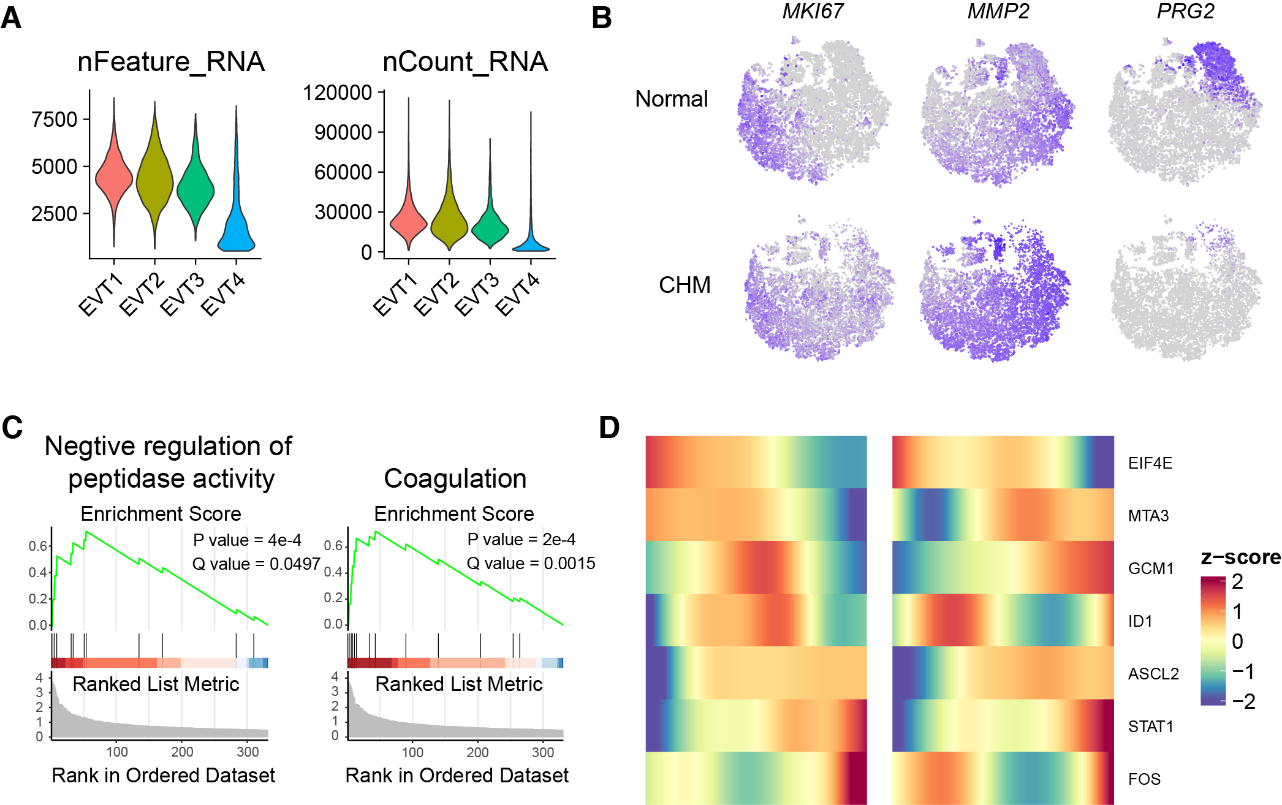


**Fig. S2** Supplementary results of EVT subtype analyses. **(A).** Violin plots depict the quality metrics of subtypes of EVT. (**B).** tSNE projection of EVT cells from normal and CHM villi, respectively, colored by the expression level of identified marker genes. (**C).** GSEA results for EVT3 markers. (**D).** Heatmap of expression levels of transcription factors smoothed by pseudotime. The values were scaled by mean-centering and transformed to a scale from -2 to 2.


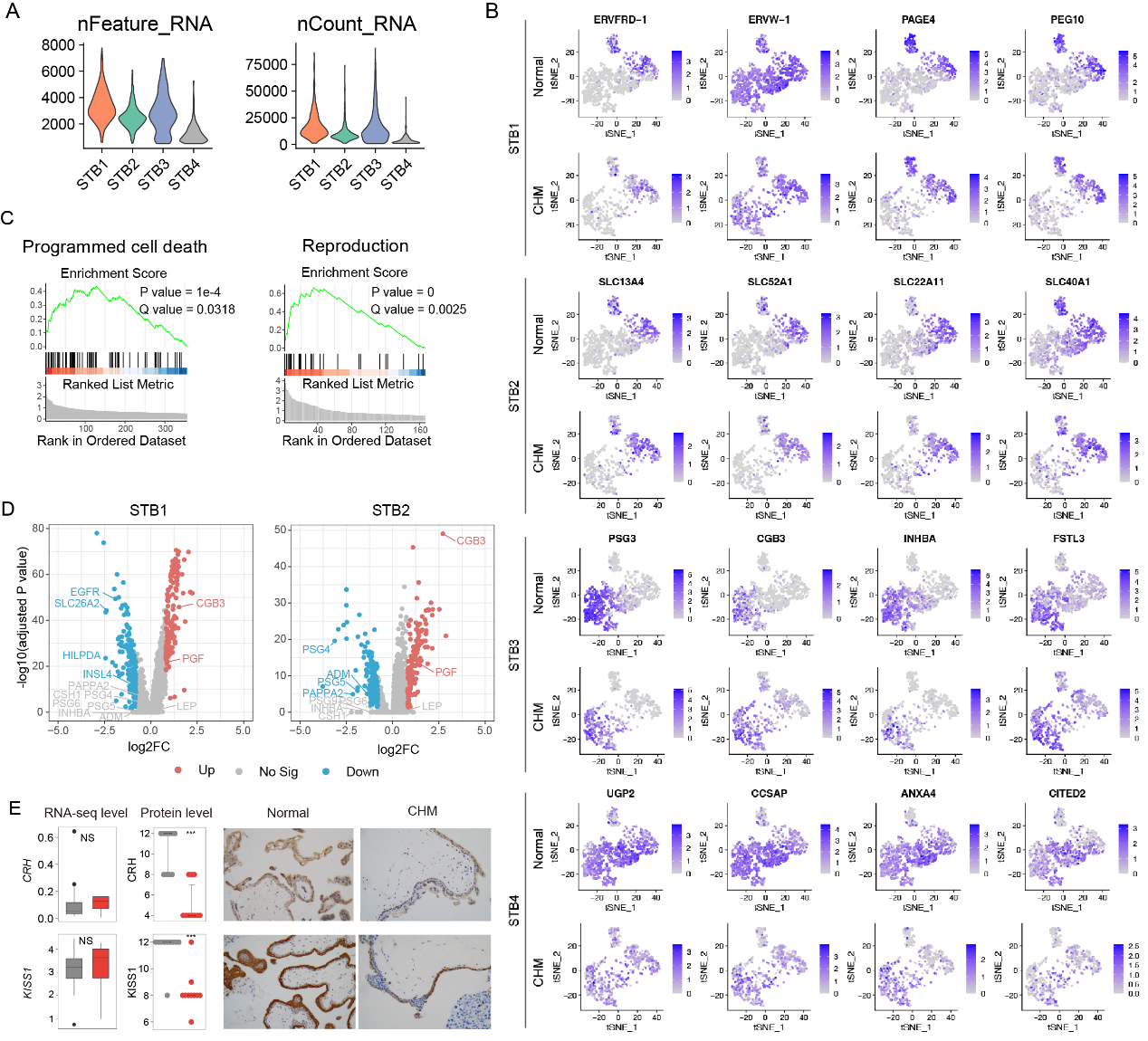


**Fig. S3** Supplementary results of STB subtype analyses. **(A)** Violin plots depict the quality metrics of subtypes of STB. (**B)** tSNE projection of STB cells from normal and CHM villi, respectively, colored by the expression level of identified marker genes. (**C)** GSEA results for STB1 markers (left) and STB3 markers (right), respectively. (**D)** Volcano plot demonstrating differentially expressed hormone-encoding genes in STB1 and STB2 cells in CHM. (**E)** Box plots showing the gene expression levels of selected hormone-encoding genes from bulk RNA-seq data (N*_normal_* = 39, N*_CHM_* = 4), and bee swarm plots of the quantified protein levels of the matched genes in the validation cohort (N*_normal_* = 10, N*_CHM_* = 10). One-way ANOVA test (***, p-value < 0.001; ****, p-value < 0.0001).
